# Association of β-cell function and cognitive impairment in patients with abnormal glucose metabolism

**DOI:** 10.1186/s12883-022-02755-6

**Published:** 2022-06-23

**Authors:** Mengyi Guo, Jiaokun Jia, Jia Zhang, Mingyue Zhou, Anxin Wang, Shengyun Chen, Xingquan Zhao

**Affiliations:** 1 Department of Neurology, Beijing Tiantan Hospital, Capital Medical University, Beijing, China; 2 Department of Neurology, Beijing Sanbo Brain Hospital, Capital Medical University, Beijing, China; 3 China National Clinical Research Center for Neurological Diseases, Beijing Tiantan Hospital, Capital Medical University, Beijing, China; 4 Department of Neurology of Shenzhen Second People’s Hospital, First Affiliated Hospital of Shenzhen University Health Science Center, Shenzhen, Guangdong China; 5 Research Unit of Artificial Intelligence in Cerebrovascular Disease, Chinese Academy of Medical Sciences, Beijing, China

**Keywords:** β-Cell function, HOMA-β, Cognitive impairment, Alzheimer’s disease (AD), Diabetes

## Abstract

**Background:**

Insulin has been demonstrated to play an important role in the occurrence and development of Alzheimer’s disease, especially in those with diabetes. β cells are important insulin-producing cells in human pancreas. This study aimed to investigate the association between β-cell dysfunction and cognitive impairment among patients over 40-year-old with abnormal glucose metabolism in Chinese rural communities.

**Methods:**

A sample of 592 participants aged 40 years or older from the China National Stroke Prevention Project (CSPP) between 2015 and 2017 were enrolled in this study. Abnormal glucose metabolism was defined when hemoglobin Alc ≥ 5.7%. Cognitive function was assessed by the Beijing edition of the Montreal Cognitive Assessment scale. Homeostasis assessment of β-cell function was performed and classified into 4 groups according to the quartiles. A lower value of HOMA-β indicated a worse condition of β-cell function. Multivariate logistic regression was used to analyze the association between β-cell function and cognitive impairment.

**Results:**

In a total of 592 patients with abnormal glucose metabolism, the average age was 60.20 ± 7.63 years and 60.1% patients had cognitive impairment. After adjusting for all potential risk factors, we found the first quartile of β-cell function was significantly associated with cognitive impairment (OR: 2.27, 95%CI: 1.32–3.92), especially at the domains of language (OR: 1.64, 95%CI: 1.01–2.65) and abstraction (OR: 2.29, 95%CI: 1.46–3.58).

**Conclusions:**

Our study showed that worse β-cell function is associated with cognitive impairment of people over 40-year-old with abnormal glucose metabolism in Chinese rural communities, especially in the cognitive domains of abstraction and language.

## Background

Alzheimer’s disease (AD), the most common type of dementia, is becoming a major challenge for global health and social care [1]. However, the present understanding of the pathogenesis of AD is limited, and there is currently no effective method for early diagnosis and improvement therapy. Aging is the most important risk factor for AD [2]. With the aggravation of population aging in China, the incidence of AD also increases rapidly, which has posed a considerable burden on society and families [3]. Mild cognitive impairment (MCI), a cognitive state between normal cognition and dementia, usually occurs at the preclinical stage of AD [4]. Patients with MCI have normal ability in daily life and only show cognitive impairment during clinical cognitive scale evaluation. Interventions in MCI stage can effectively alleviate the progression of cognitive deficiency in AD, and improve the prognosis and quality of patients’ life. According to many previous studies [5–7], diabetes is one of the adjustable risk factors of MCI, which has been shown in Chinese rural communities in our previous study [8]. There are 12.8% of Chinese adults diagnosed as diabetes, which is currently found as the largest proportion of adults affected by diabetes of any country [9]. The high incidence of diabetes may further adversely aggravate the progression of AD. Therefore, exploring the mechanisms under the association between diabetes and cognitive impairment, exploring effective and timely interventions applied for MCI stage, could significantly prevent the prevalence of AD.

β-cell dysfunction, which means the inability of the pancreas to produce enough insulin in response to glucose stimulation, is a typical pathological mechanism in type 2 diabetes mellitus (T2DM) [10]. Insulin, as well as insulin-related growth factors, plays an important role in the development of central nervous system (CNS) [11, 12]. Impaired signaling through insulin/insulin-like growth factor (IGF) receptors in the brain can damage a range of neural and glial functions, including glucose homeostasis, energy metabolism, and white matter fiber structure and function [13, 14]. As reported, dysfunction of insulin signaling acts the core of the neurodegenerative cascade in AD [13, 14]. AD-associated abnormalities in energy metabolism are also reported to be caused by insulin resistance or reduced insulin actions in the brain [15]. Thus, AD is called as “type 3 diabetes”, which means a type of brain-specific or brain-restricted diabetes [16]. Based on the correlation between impaired insulin signaling and AD, β-cell dysfunction, an important reason for insulin deficiency in brain, may also have adverse effects on cognitive function. Furtherly researching the relationship between β-cell dysfunction and MCI in people with prediabetes or diabetes, will help delay the progression of cognitive impairment, as well as reduce the incidence of AD.

In rural areas of China, due to the more serious condition of population aging and the relatively weak awareness of medical treatment, the control and therapy of diabetes-related cognitive impairment are significantly impeded. Thus, to provide evidence for the prevention and management of cognitive dysfunction in Chinese rural communities, this study was performed to investigate the association between β-cell dysfunction and cognitive decline in people (≥40 years old) with abnormal glucose metabolism. And the study hypothesis was that the β-cell dysfunction may act as a risk factor of cognitive impairment in the specific population of China.

## Methods

### Study design and population

The China National Stroke Prevention Project (CSPP) is a community-based, prospective, long-term follow-up study to promote the management of stroke in Chinese adults. It is supported by the China Ministry of Health and China Ministry of Finance. The protocol of subject enrollment for the CSPP study has been described previously [17]. Residents who aged≥40 years, belonged to the selected communities or lived there for over half a year were invited to join a screening program. The questionnaire, clinical examination and laboratorial evaluation were performed at baseline. This study cohort was a sub-population of 5188 subjects residing in the Beiqijia community and Shunyi district hospital of Beijing between September 2015 and September 2017. Ultimately, we excluded 4596 participants according to the exclusion criteria, and 592 participants were enrolled. The exclusion criteria were shown as follows: (1) with normal glucose metabolism; (2) with history of neurological disease which may affect the cognitive ability, such as transient ischemic attacks (TIA) and stroke; (3) existing factors which may interfere cognitive assessment and other examination, such as hearing or visual impairment, difficulties to cooperate, and mental disorder; (4) with incomplete baseline information.

### Assessment of cognitive function

The cognitive function was evaluated by the Beijing edition of the Montreal Cognitive Assessment scale (MoCA). MoCA is a screening tool with high sensitivity and specificity in detecting MCI [18]. It consists of seven cognitive domains including visuospatial/executive functions (5 points), naming (3 points), language (3 points), attention (6 points), abstraction (2 points), delayed recall (5 points) and orientation (6 points). The score of MoCA ranges from 0 to 30, and the total score (if< 30 points) needs to be added 1 point when the subject is educated 12 years or less. According to the original explanation of MoCA results, the highest score was 30, and cognitive impairment was defined when a corrected MoCA score < 26. In this study, all staff responsible for the MoCA test were systematically trained and evaluated by the CSPP.

### Assessment of FPI, FPG and other biochemical indexes

Fasting plasma glucose (FPG) was measured by glucometer and glucose test strips (ACCU-CHEKR Active, Roche Diabetes Care GmbH, German) after an overnight fast of the participants. At the same time, superficial venous blood was collected for measurement of the fasting insulin and other biochemical indices. The fasting plasma insulin (FPI) was tested by an automatic light-emitting analyzer (DXI800, Beckman, American), with the technology of microparticle chemiluminescence immunoassay. All blood samples were collected in EDTA tubes and measured with an automated analyzer (Hitachi 747; Hitachi, Tokyo, Japan) in the laboratory at Beiqijia and Shunyi Hospital.

According to the guidelines from the Standards of Medical Care in Diabetes-2019, the abnormal glucose metabolism was defined when hemoglobin Alc (HbAlc) ≥ 5.7% [19].

### Assessment of the index of β-cell function and insulin resistance

The homeostasis model assessment of β-cell function (HOMA-β) was used to evaluate the β-cell function. HOMA-β is an indicator of pancreatic β-cell function based on FPI and FPG levels, which was estimated as follows: HOMA-β = FPI level (μU/ml) × 20/[FPG (mM)-3.5]. For HOMA-β, the lower value means worse β-cell function. In this study, patients were grouped by the quartile of HOMA-β (Q1: HOMA-β ≤ 36.69, Q2: 36.70 ≤ HOMA-β ≤ 56.58, Q3: 56.59 ≤ HOMA-β ≤ 83.29, Q4: HOMA-β ≥ 83.30). The Homeostasis model assessment of insulin resistance (HOMA-IR) was calculated as follows: HOMA-IR = FPI level (μU/ml) x FPG (mM)/22.5 [20, 21]. For HOMA-IR, the higher value indicates a more serious degree of insulin resistance.

### Questionnaire of baseline information

Our questionnaire included demographic information (such as age, gender, and education level), lifestyle risk factors (such as smoking and alcohol consumption), medical history (high blood pressure, diabetes, and dyslipidemia), family history, and other necessary information. All relevant staff have been trained and evaluated by CSPP. All questionnaire data were entered into the EpiData 3.0 as an electronic database by two trained staff. Standardized procedures are followed by experienced data managers to check data for integrity and potential errors.

Education level was defined as “elementary education or below”, “middle school education” and “high school education or above”. Age groups were categorized as “40-59 years old” and “≥60 years old”. Body mass index (BMI) was classified into “≥ 25” and “< 25” [22]. Alcohol consumption and smoking were both categorized as “never” or “former or current”, based on the self-provided information of patients. Hypertension was defined when systolic blood pressure ≥ 140 mmHg or diastolic blood pressure ≥ 90 mmHg, or with a self-reported history of hypertension. Hyperhomocysteinemia (High-Hcy) was diagnosed by serum homocysteine (> 15 μmol/L). Dyslipidemia was diagnosed when low-density lipoprotein cholesterol (LDL-C) ≥ 3.37 mmol/L, or high-density lipoprotein cholesterol (HDL-C) < 1.04 mmol/L, or triglycerides (TG) ≥ 1.7 mmol/L, or total cholesterol (TC) ≥ 5.17 mmol/L, or with a self-reported history of dyslipidemia [23].

### Statistical analysis

Statistical analysis was performed using the IBM SPSS Statistics 23.0. All statistical analyses were two-tailed, and the *P* value less than 0.05 was considered statistically significant.

During univariate analysis, we used analysis of variance (ANOVA) for normally distributed variables, Wilcoxon or Kruskal–Wallis tests for non-normally distributed variables, and χ2 test for the classification variables. Multivariate logistic regression analysis was used to analyze the relationship between β-cell function and cognitive impairment. The covariates with *P* < 0.1 in univariate analysis were collected for multivariate models. Some additional variables (suggested to be risk factors of cognitive impairment in previous studies) were also included [24, 25]. Besides, all potential variables were evaluated in subgroups, to assess whether there was any significant interaction between these variables and the relationship between β-cell function and the prevalence of cognitive impairment.

Multivariate ordinal and binary logistic regression analysis were used to analyze the association between β-cell function and specific cognitive domains. After parallel line checking, visuospatial/executive functions, naming, attention, language, and abstraction and recall were found suitable for ordinal logistic regression, but the orientation domain was not. Therefore, the orientation was analyzed by binary logistic regression. For analysis, the orientation was divided into two groups: the maximum was one group, and the rest were the other group.

## Results

### Baseline characteristics

The mean age of the 592 participants was 60.20 ± 7.63 years, ranging from 40 years to 85 years; 181 (30.6%) were men, and 152 (25.7%) of them had high school education or above. Out of 592 patients, 356 (60.1%) had cognitive impairment. For the entire sample, the mean HOMA-IR was 2.27 ± 2.03, and the mean MoCA score was 23.77 ± 4.27.

Baseline characteristics stratified by the quartile of HOMA-β were shown in Table 1. Patients with higher HOMA-β were more likely to be female, and have higher BMI and lower HDL-C (*P* < 0.05, Table 1). Insulin resistant levels were different among different quartiles, but the trend was not clear.

**Table 1:** Baseline characteristics of the study population stratified by the quartile of HOMA-β

Variables		HOMA-β				P
	Total (*n* = 592)	Q1 (*n* = 148)	Q2 (*n* = 148)	Q3 (*n* = 148)	Q4 (*n* = 148)	
Age (years)	60.20 ± 7.63	61.56 ± 7.65	60.07 ± 7.06	59.67 ± 7.52	59.48 ± 8.16	0.079
Male, n (%)	181 (30.6)	57 (38.5)	44 (29.7)	47 (31.8)	33 (22.3)	0.025*
BMI (kg/m^2^), n (%)						<0.001*
≥ 25	429 (72.5)	93 (62.8)	101 (68.2)	112 (75.7)	123 (83.1)	
< 25	163 (27.5)	55 (37.2)	47 (31.8)	36 (24.3)	25 (16.9)	
Education level, n (%)						0.073
Primary education or below	83 (14.0)	27 (18.2)	14 (9.5)	15 (10.1)	27 (18.2)	
Elementary education	357 (60.3)	85 (57.4)	89 (60.1)	100 (67.6)	83 (56.1)	
High school education or above	152 (25.7)	36 (24.3)	45 (30.4)	33 (22.3)	38 (25.7)	
Smoking, n (%)						0.662
Never	488 (82.4)	118 (79.7)	121 (81.8)	123 (83.1)	126 (85.1)	
Former or current	104 (17.6)	30 (20.3)	27 (18.2)	25 (16.9)	22 (14.9)	
Drinking, n (%)						0.231
Never	478 (80.7)	112 (75.7)	120 (81.1)	120 (81.1)	126 (85.1)	
Former or current	114 (19.3)	36 (24.3)	28 (18.9)	28 (18.9)	22 (14.9)	
HDL-C (mmol/L)	1.39 ± 0.32	1.43 ± 0.35	1.45 ± 0.31	1.38 ± 0.32	1.31 ± 0.30	< 0.001*
LDL-C (mmol/L)	3.32 ± 0.91	3.36 ± 1.03	3.45 ± 0.93	3.29 ± 0.83	3.19 ± 0.82	0.150
Dyslipidemia, n (%)	456 (77.0)	115 (77.7)	107 (72.3)	112 (75.7)	122 (82.4)	0.212
Hypertension, n (%)	256 (43.2)	61 (41.2)	55 (37.2)	65 (43.9)	75 (50.7)	0.120
Heart disease, n (%)	78 (13.2)	25 (16.9)	13 (8.8)	17 (11.5)	23 (15.5)	0.146
High-Hcy, n (%)	166 (28.0)	38 (25.7)	43 (29.1)	42 (28.4)	43 (29.1)	0.903
Cognitive Impairment, n (%)	356 (60.1)	102 (68.9)	89 (60.1)	84 (56.8)	81 (54.7)	0.064
HOMA-IR	2.27 ± 2.03	1.81 ± 1.44	1.71 ± 0.94	2.09 ± 1.13	3.48 ± 3.22	<0.001*

*BMI* body mass index, *HDL-C* high density lipoprotein cholesterol, *LDL-C* low density lipoprotein cholesterol, *High-Hcy* high homocysteine, *HOMA-IR* homeostasis assessment model, insulin resistance, *HOMA-β* homeostasis assessment model, β-cell function

*statistically significant

### Correlation between β-cell function and cognitive impairment

Table 2 showed the association between HOMA-β and cognitive impairment. In univariate analysis (OR: 1.83, 95%CI: 1.14–2.95, *P* = 0.012), as well as in multivariate logistic regression adjusting for age, and sex and education in Model 1 (OR: 1.81, 95%CI: 1.10–2.99, *P* = 0.020), the first quartile was associated with cognitive impairment. After further adjusting for BMI, smoking, alcohol assumption, hypertension, dyslipidemia, heart disease, high-hcy and HOMA-IR in Model 2, the significance of the association between the first quartile with cognitive impairment remained (OR: 2.27, 95%CI: 1.32–3.92, *P* = 0.003).

**Table 2:** Multivariate binary logistic regression analysis for the association between HOMA-β and cognitive impairment

HOMA-β	Univariate analysis		Model 1		Model 2	
	OR (95% CI)	P	OR (95% CI)	P	OR (95% CI)	P
Q4	Ref.		Ref.		Ref.	
Q3	1.09 (0.69–1.72)	0.726	1.11 (0.69–1.80)	0.660	1.26 (0.76–2.09)	0.370
Q2	1.25 (0.79–1.98)	0.347	1.41 (0.87–2.28)	0.170	1.66 (0.98–2.79)	0.060
Q1	1.83 (1.14–2.95)	0.012*	1.81 (1.10–2.99)	0.020*	2.27 (1.32–3.92)	0.003*

*HOMA-β* homeostasis assessment model, β-cell function, *Ref* reference, *95%CI* 95% confidence interval, *OR* odds ratios

*, statistically significant

Model 1: adjusted for age, sex, and education;

Model 2: adjusted for age, sex, education, BMI, smoking, alcohol use, hypertension, dyslipidemia, heart disease, high-hcy, and HOMA-IR

Stratified analyses showed that age, gender, BMI, education, smoking, alcohol assumption, hypertension, dyslipidemia, heart disease and the prevalence of high-hcy had no interaction effect with β-cell function on cognitive impairment (*P* > 0.05, Table 3).

**Table 3:** Multivariate analysis for the association between HOMA-β and cognitive impairment stratified by other sociodemographic characteristics

Variables	HOMA-β							P for interaction
	Q4	Q3		Q2		Q1		
	OR (95%CI)	OR (95%CI)	P	OR (95%CI)	P	OR (95%CI)	P	
Age (years old)								0.613
< 60	Ref.	1.70 (0.85–3.39)	0.133	2.02 (1.00–4.09)	0.052	2.30 (1.08–4.91)	0.032*	
≥ 60	Ref.	0.86 (0.40–1.86)	0.700	1.23 (0.55–2.75)	0.613	2.18 (0.96–4.96)	0.064	
Gender								0.951
Male	Ref.	1.23 (0.43–3.58)	0.700	1.38 (0.47–4.03)	0.550	2.25 (0.78–6.45)	0.133	
Female	Ref.	1.23 (0.68–2.22)	0.497	1.77 (0.96–3.26)	0.067	2.28 (1.18–4.40)	0.015*	
BMI (Kg/m2), n (%)								0.313
< 25	Ref.	0.69 (0.21–2.30)	0.544	0.83 (0.26–2.68)	0.757	2.03 (0.61–6.75)	0.246	
≥ 25	Ref.	1.59 (0.89–2.83)	0.119	2.17 (1.19–3.97)	0.012*	2.11 (1.13–3.96)	0.020*	
Education level								0.383
Primary education or below	Ref.	0.34 (0.06–2.10)	0.245	1.54 (0.18–13.20)	0.695	0.85 (0.14–5.00)	0.856	
Elementary education	Ref.	1.64 (0.87–3.10)	0.130	2.24 (1.14–4.41)	0.020*	2.80 (1.38–5.68)	0.004*	
High school education or above	Ref.	0.89 (0.32–2.53)	0.832	0.86 (0.31–2.37)	0.775	1.56 (0.51–4.73)	0.433	
Smoking								0.989
Never	Ref.	1.27 (0.73–2.21)	0.396	1.60 (0.91–2.83)	0.104	2.27 (1.24–4.13)	0.008*	
Former or current	Ref.	1.39 (0.32–6.07)	0.666	1.86 (0.42–8.19)	0.412	2.02 (0.48–8.47)	0.339	
Drinking								0.644
Never	Ref.	1.29 (0.74–2.24)	0.365	1.88 (1.06–3.34)	0.031*	2.85 (1.54–5.28)	0.001*	
Former or current	Ref.	0.73 (0.16–3.39)	0.692	0.89 (0.21–3.91)	0.882	0.85 (0.21–3.49)	0.825	
Hypertension								0.772
No	Ref.	1.38 (0.69–2.79)	0.363	1.64 (0.81–3.30)	0.170	2.45 (1.17–5.15)	0.018*	
Yes	Ref.	0.95 (0.44–2.04)	0.897	1.38 (0.61–3.14)	0.441	1.91 (0.83–4.40)	0.131	
Dyslipidemia								0.228
No	Ref.	3.28 (0.89–12.09)	0.075	5.00 (1.25–20.08)	0.023*	4.67 (1.08–20.18)	0.039*	
Yes	Ref.	1.07 (0.61–1.89)	0.804	1.35 (0.76–2.43)	0.310	1.92 (1.05–3.51)	0.034*	
Heart disease								0.938
No	Ref.	1.17 (0.68–2.01)	0.571	1.47 (0.84–2.55)	0.174	2.00 (1.12–3.58)	0.020*	
Yes	Ref.	2.54 (0.48–13.54)	0.274	4.03 (0.62–26.18)	0.144	9.84 (1.40–69.28)	0.022*	
High-Hcy								0.420
No	Ref.	1.53 (0.85–2.77)	0.158	1.92 (1.04–3.54)	0.037*	2.20 (1.18–4.14)	0.014*	
Yes	Ref.	0.76 (0.26–2.20)	0.612	1.36 (0.46–4.03)	0.576	3.42 (0.94–12.45)	0.062	

*HOMA-β* homeostasis assessment model, β-cell function, *BMI* body mass index, *High-Hcy* high homocysteine

*statistically significant

### Correlation between β-cell function and specific cognitive domains

Table 4 showed the differences of seven specific cognitive domains among 4 quartiles of HOMA-β, respectively. The function of abstraction and language were better in patients with higher HOMA-β (*P* < 0.05, Table 4). After adjusting for all potential covariates, the first quartile was further significantly related with impairment of language (OR: 1.64, 95%CI: 1.01–2.65, *P* = 0.045) and abstraction (OR: 2.29, 95%CI: 1.46–3.58, *P* < 0.001). The results were shown in Table 5.

**Table 4:** Scores of specific domains of the study population stratified by HOMA-β

Variables	Total	HOMA-β				P
		Q1	Q2	Q3	Q4	
Visuospatial/Executive functions	2.82 ± 1.22	2.76 ± 1.25	2.67 ± 1.23	2.99 ± 1.19	2.84 ± 1.19	0.130
Naming	2.70 ± 0.56	2.70 ± 0.59	2.70 ± 0.59	2.74 ± 0.47	2.67 ± 0.59	0.722
Attention	5.12 ± 1.12	5.03 ± 1.21	5.08 ± 1.20	5.20 ± 1.00	5.15 ± 1.07	0.583
Language	2.49 ± 0.74	2.39 ± 0.80	2.42 ± 0.75	2.59 ± 0.68	2.57 ± 0.71	0.021*
Abstraction	1.19 ± 0.79	1.05 ± 0.80	1.18 ± 0.80	1.24 ± 0.73	1.29 ± 0.79	0.048*
Memory	2.66 ± 1.84	2.47 ± 1.84	2.72 ± 1.90	2.64 ± 1.76	2.80 ± 1.88	0.441
Orientation	5.85 ± 0.52	5.84 ± 0.54	5.89 ± 0.45	5.82 ± 0.58	5.87 ± 0.51	0.724

*HOMA-β* homeostasis assessment model, β-cell function

*statistically significant

**Table 5:** Multivariate analysis for the association of HOMA-β and specific cognitive domains

Variables	HOMA-β						
	Q4	Q3		Q2		Q1	
	OR (95%CI)	OR (95%CI)	P	OR (95%CI)	P	OR (95%CI)	P
Visuospatial/Executive functions	Ref.	0.78 (0.51–1.18)	0.233	1.38 (0.91–2.10)	0.131	1.21 (0.79–1.84)	0.382
Naming	Ref.	0.96 (0.56–1.66)	0.895	1.03 (0.59–1.77)	0.927	0.97 (0.55–1.68)	0.901
Attention	Ref.	1.04 (0.67–1.61)	0.870	1.23 (0.79–1.92)	0.351	1.27 (0.81–1.97)	0.296
Language	Ref.	0.96 (0.59–1.58)	0.886	1.70 (1.05–2.75)	0.030*	1.64 (1.01–2.65)	0.045*
Abstraction	Ref.	1.40 (0.90–2.16)	0.136	1.67 (1.07–2.59)	0.024*	2.29 (1.46–3.58)	< 0.001*
Memory	Ref.	1.36 (0.90–2.05)	0.141	1.25 (0.83–1.89)	0.291	1.39 (0.91–2.11)	0.126
Orientation	Ref.	1.33 (0.62–2.86)	0.458	0.95 (0.42–2.17)	0.904	1.12 (0.51–2.44)	0.779

*Ref* reference, *95%CI* 95% confidence interval, *OR* odds ratios

*statistically significant

## Discussion

In our study, lower β-cell function defined by HOMA-β was found associated with an increased risk of cognitive impairment (especially in language and abstraction) in middle aged and elderly people (≥40 years) with abnormal glucose metabolism in Chinese rural communities. β cells are important insulin-secreting cells in human islet. The dysfunction of them may lead to significant insulin deficiency and impaired insulin signaling in brain. A lot of studies have shown that dysfunction of insulin signaling forms the core of neurodegeneration in Alzheimer’s disease (AD) [13, 26, 27]. β-cell dysfunction is common in people with prediabetes or diabetes [10]. Thus, great attention should be paid to the β-cell dysfunction related cognitive impairment in middle-aged and old people with abnormal glucose metabolism.

There are some potential mechanisms that may explain the relationship between β-cell dysfunction and cognitive impairment. Firstly, the insulin deficiency caused by β-cell dysfunction can influence cognition by affecting brain energy metabolism. Studies have reported that in the early stages of AD, cerebral glucose utilization may reduce by up to 45% [28]. And the decrease in brain energy metabolism predates the cognitive decline. It is also reported that cerebral glucose metabolism increased after the restoration of basal insulin levels in metabolically healthy participants [29]. Besides, insulin deficiency also damages vascular function through effects on vasoreactivity, lipid metabolism, and inflammation [30]. As reported, lower β-cell function may lead to elevated intima-media thickness [31] and increased risk of recurrent stroke [32], both of which have been found associated with cognitive impairment [33, 34]. In addition, insulin plays an important role in synaptic plasticity of hippocampus. In insulin-deficient diabetic rats, hippocampal synaptic plasticity such as long-term enhancement (LTP) was found inhibited [35]. Insulin can also modulate some neurotransmitters in the brain that are involved in cognitive function, such as N-methyl-D-aspartate (NMDA) [36], acetylcholine and norepinephrine [37, 38]. Based on the importance of insulin in AD, transnasal insulin therapy is suggested for AD therapy now. The therapeutic effects have also been demonstrated in some clinical trials of patients [39], as well as in animal models [40].

In people of rural communities, which account for 50.32% of the Chinese population [8], due to the large and aging population, and relatively backward medical level, there may exist some difficulties in the screening and therapy of initial β-cell dysfunction and MCI. Therefore, promoting early screening of those abnormalities, and searching for convenient and accurate screening methods, are necessary for the arrangement of β-cell dysfunction related dementia in old people of Chinese rural communities. In this study, the MoCA scale was used to test the cognitive function, and HOMA-β was used to evaluate the function of β cells. Compared with some complex scales, MoCA is more easily promoted and operated by physicians, and more comprehensible for old people. In addition, MoCA has higher sensitivity and specificity in the detection of MCI [41]. HOMA is a mathematical model for calculating the steady-state glucose and insulin concentrations. Compared to the hyperglycemic clamp technique, the gold standard for evaluation of β-cell function, HOMA-β is more inexpensive and easier to operate. Moreover, HOMA-β was well correlated with hyperglycemic clamp under different glucose tolerance conditions [21]. Thus, due to their convenience and accuracy, the method of MoCA scale and HOMA-β can be widely used in rural communities in China, as well as some clinical large-scale epidemiological studies.

Notably, the influence of insulin resistance was corrected in the exploration of correlation between β-cell dysfunction and cognitive impairment. Insulin resistance is defined as a failure of target tissues to exhibit a normal response to insulin [42]. It usually coexists with β-cell dysfunction in diabetic patients [43]. Since the influence of insulin resistance on the value of FPG and FPI, HOMA-β may underestimate the decline of β-cell function [44]. In our study, after adjustment for HOMA-IR, the association of β-cell dysfunction and cognitive impairment remained. This result suggests that, in people with dysglycemia of Chinese rural communities, the association between insulin secretion and cognitive impairment is independent of insulin sensitivity.

In addition, in this study, the impairment of language was found associated with β-cell function in people over 40-year-old with abnormal glucose metabolism. For old diabetic patients, language function was particularly important for their social functioning, which was closely associated with the psychological states and disease management. The elderly with language dysfunction may fail to communicate well with their families or doctors, resulting in emotional distress and some diseases due to delayed detection. As time goes by, patients may develop diabetes aggravation, depression, and some related complications, such as dyslipidemia and hypertension [45]. Therefore, timely detection and therapy of β -cell disorder can effectively improve the quality of life of old diabetic patients.

There were also some limitations in this study. First, large parts of patients in our study with missing data were excluded from the current analysis. The effect of this on the results is unclear, although the differences between baseline characteristics of included and excluded patients are not statistically significant (data not shown). Second, due to the large missing data, the detailed medication information was not included in the final analysis in this study, which was important for diabetes management and should be furtherly analyzed in the further studies. Third, due to the lack of follow-up data, the measurements of the influence of β-cell function on subsequent cognitive function in several years after baseline were unavailable. Further research with a large sample size and a long-term follow-up is required to examine the association between β-cell function and cognitive impairment in people with abnormal glucose metabolism of Chinese rural communities.

## Conclusions

β-cell dysfunction may be an independent risk factor of cognitive impairment, especially in the domains of language and abstraction, among the middle-aged and elderly population (≥40 years) with abnormal glucose metabolism in rural communities of China.

## Data Availability

The datasets used and analyzed during the current study are available from the corresponding author on reasonable request.

## Abbreviations

AD: Alzheimer’s disease
MCI: Mild cognitive impairment
T2DM: Type 2 diabetes mellitus
CNS: Central nervous system
IGF: Insulin-like growth factor
TIA: Transient ischemic attacks
MoCA: Montreal Cognitive Assessment scale
FPG: Fasting plasma glucose
FPI: Fasting plasma insulin
HbAlc: Hemoglobin Alc
HOMA-IR: Homeostasis assessment model, insulin resistance
HOMA-β: Homeostasis assessment model, β-cell
High-Hcy: Hyperhomocysteinemia
LDL-C: Low-density lipoprotein cholesterol
HDL-C: High-density lipoprotein cholesterol
TG: Triglycerides
TC: Total cholesterol
ANOVA: Analysis of variance
BMI: Body mass index
LTP: Long-term enhancement
NMDA: N-methyl-D-aspartate
